# An ShRNA Based Genetic Screen Identified Sesn2 as a Potential Tumor Suppressor in Lung Cancer via Suppression of Akt-mTOR-p70S6K Signaling

**DOI:** 10.1371/journal.pone.0124033

**Published:** 2015-05-11

**Authors:** Hui Xu, Huiying Sun, Haiyuan Zhang, Jiawei Liu, Fangfang Fan, Yilan Li, Xuelian Ning, Yue Sun, Shaochun Dai, Baogang Liu, Min Gao, Songbin Fu, Chunshui Zhou

**Affiliations:** 1 The Laboratory of Medical Genetics, Harbin Medical University, Harbin, China; 2 The Tumor Hospital, Harbin Medical University, Harbin, China; 3 The Fourth Affiliated Hospital, Harbin Medical University, Harbin, China; New York University School of Medicine, UNITED STATES

## Abstract

**Background:**

Lung cancer is emerging rapidly as the leading death cause in Chinese cancer patients. The causal factors for Chinese lung cancer development remain largely unclear. Here we employed an shRNA library-based loss-of-function screen in a genome-wide and unbiased manner to interrogate potential tumor suppressor candidates in the immortalized human lung epithelial cell line BEAS-2B.

**Methods/Results:**

Soft agar assays were conducted for screening BEAS-2B cells infected with the retroviral shRNA library with the acquired feature of anchorage-independent growth, large (>0.5mm in diameter) and well—separated colonies were isolated for proliferation. PCRs were performed to amplify the integrated shRNA fragment from individual genomic DNA extracted from each colony, and each PCR product is submitted for DNA sequencing to reveal the integrated shRNA and its target gene. A total of 6 candidate transformation suppressors including INPP4B, Sesn2, TIAR, ACRC, Nup210, LMTK3 were identified. We validated Sesn2 as the candidate of lung cancer tumor suppressor. Knockdown of Sesn2 by an shRNA targeting 3’ UTR of Sesn2 transcript potently stimulated the proliferation and malignant transformation of lung bronchial epithelial cell BEAS-2B via activation of Akt-mTOR-p70S6K signaling, whereas ectopic expression of Sens2 re-suppressed the malignant transformation elicited by the Sesn2 shRNA. Moreover, knockdown of Sesn2 in BEAS-2B cells promoted the BEAS-2B cell-transplanted xenograft tumor growth in nude mice. Lastly, DNA sequencing indicated mutations of Sesn2 gene are rare, the protein levels of Sesn2 of 77 Chinese lung cancer patients varies greatly compared to their adjacent normal tissues, and the low expression level of Sesn2 associates with the poor survival in these examined patients by Kaplan Meier analysis.

**Conclusions:**

Our shRNA-based screen has demonstrated Sesn2 is a potential tumor suppressor in lung epithelial cells. The expression level of Sesn2 may serve as a prognostic marker for Chinese lung cancer patients in the clinic.

## Introduction

Lung cancer is emerging as the most common and deadly malignancy in China as well as in the world [1,2]. Based on pathological features, lung cancer can be divided into two major subtypes, non-small-cell lung carcinoma (NSCLC) and small cell lung carcinoma (SCLC). NSCLC that accounts for more than 80% of all lung cancer cases can be further divided into adenocarcinoma (~48%), squamous cell carcinoma (~28%) and large cell carcinoma (~24%) [1,3]. Despite the great advances achieved in the diagnostics, surgical operation, radiotherapy and targeted therapies, lung cancer still holds a quite poor prognosis and its 5 year survival rate remains as low as 10%-15% in the past 30 years [3].

The mechanisms driving lung cancer development are complex, genetic alterations, smoking and various environmental pollutions are common causal factors attributed to lung cancer occurrence. Tumor suppressors with loss-of-function mutations, deletions, and/or epigenetic silencing often play a crucial role in lung tumorigenesis [4]. For example, the mutation rate of p53 gene in non-small cell lung cancer (NSCLC) can reach to 60%, even goes up to 80% in small cell lung cancer (SCLC) [5]. Other tumor suppressors such as PTEN with much lower mutation rate also involve in lung adenocarcinoma [6]. In addition to better understanding the molecular alterations occurred during lung cell malignant transformation, discovery of lung cancer related tumor suppressor genes also provides more effective and personalized therapies for lung cancer treatment [7]. To this end, to identify novel tumor suppressors in a genome-wide and unbiased manner is one of the central tasks for lung cancer research. However, identifying the new tumor suppressor genes is rather difficult due to their recessive expression nature. Cancer whole genomic analysis indicates that there are many low ratio mutations in the tumor cells, and the mutations vary between different origins of tissues [8]. An shRNA library-based loss-of-function screen targeting human transcriptome to interrogate potential tumor suppressor candidates systematically in immortalized human cells has been proven to be a powerful approach for identification of new tumor suppressors [9,10], by using this approach, a number of new tumor suppressors including Rest, PTPN12, etc. were discovered [11,12].

The Sestrins belong to a small and evolutionary conserved family composed of three members in mammals, of which Sesn1 and Sesn2 are stress inducible and p53 regulated [13,14]. The ability of Sesn1 and Sesn2 to inhibit cell growth and proliferation was attributed to their inhibition of mTORC1 activity through an AMPK dependent mechanism in a variety of human and mouse cell lines, as well as in mouse liver [15].

The consequence of Sesn2 in control of cell growth and survival remains controversial. The exact role of Sesn2 on cell survival might depend on the nature of the stress. It has been shown that Sesn2 expression inhibits cell growth and proliferation in response to genotoxic stress (such as DNA damage) and Sesn2 deficiency renders murine fibroblast more susceptible to oncogenic transformation via the relief of p53 dependent inhibition of mTOR [16]. Moreover, elevated Sesn2 inhibited IR-induced mTOR signalling and sensitized MCF7 cells to IR irradiation [17], in contrast, the expression of Sesn2 protected ischemia, low glucose and H_2_O_2_ induced apoptosis in LNCaP cells [18].

## Results

### shRNA screen for transformation suppressors of lung epithelial cells

**Fig 1:**
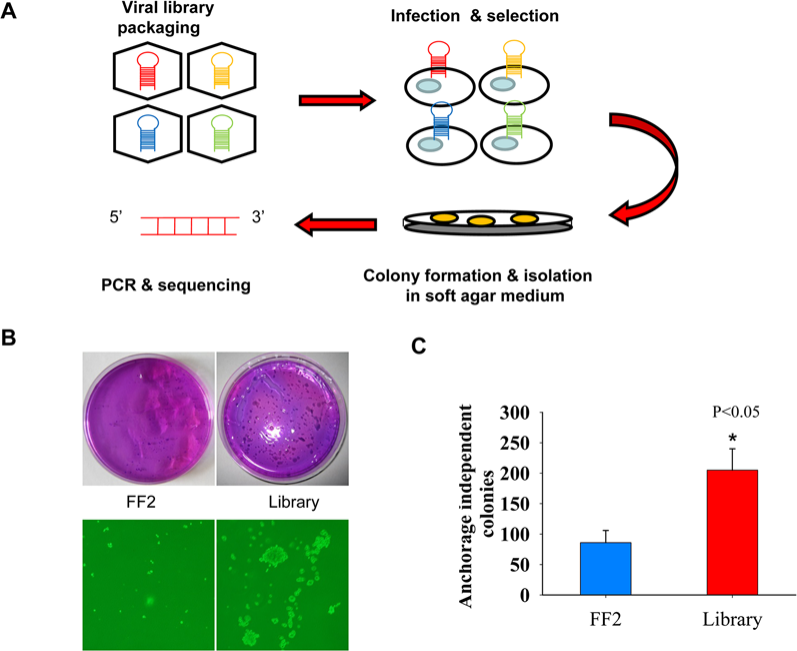
Identification of tumor suppressor genes in immortalized human bronchial epithelial BEAS-2B cells. **(A)** A schematic diagram of a genetic screen for transformation suppressors of BEAS-2B cells. Retroviruses carrying the shRNA library were generated by transfection of viral packaging cells with shRNA plasmids. BEAS-2B cells were infected with FF2 shRNA or shRNA library viruses and cultured in soft agar in 10cm plates for 3 weeks, large and single colonies were isolated for cell expansion, PCR amplification and DNA sequencing of the integrated shRNAs. **(B)** Representative images of colonies formed on soft agar plates. Transformed colonies were observed by crystal violet staining (upper panel) or under light microscopy at 20x magnification (lower panel). **(C)** More and larger anchorage independent colonies were formed in library infected cells than in shFF2 infected cells, data are presented as average colony number ± standard deviation per plate based on three independent infection experiments, *p<0.05 by the Student’s t-test.

Anchorage independent growth is a hallmark of cellular malignant transformation *in vitro*. The transformed cells lose the contact inhibition, can grow and survive without attaching themselves to the culture matrix. Therefore, soft agar assay to measure the anchorage independent growth is a useful method to find new transformation suppressor genes [19]. As most lung cancer arising from epithelial cells, we chose the bronchial epithelial cells BEAS-2B for the primary screen. BEAS-2B cells derived from healthy human lung tissue were transformed with SV40-Large T antigen. These cells are immortalized but lack of ability to proliferate efficiently in the absence of extracellular matrix and form tumors in nude mice [20]. We infect BEAS-2B cells with a retroviral shRNA library constructed in pSM2 retroviral vector. The shRNA library consists of 87,283 shRNAs targeting 32,216 human transcripts [9,10]. The library was screened in 72 pools with 1,000 shRNAs per pool using a protocol described previously (Fig 1A) [21]. The infected BEAS-2B cells were selected with 0.5μg/mL puromycin for 1 week, then, we assessed the infected cells for anchorage-independent proliferation (Fig 1B). Both the negative control shFF2 and shRNA library infected BEAS-2B cells were grown in 0.35% top agar for three weeks. We counted colony numbers from the shFF2 control and shRNA library plates by violet crystal staining. Whereas shFF2 cells induced much smaller and fewer numbers of colony, the library transformed cells elicited much more and larger colonies in the upper soft agar medium. The average numbers of transformed colony were 86±20 per 10cm plate for shFF2 cells and 205±35 for shRNA library cells based on results from three independent experiments (Fig 1C). The large (>0.5mm in diameter) and well-separated single colonies were picked for expansion in regular monolayer culture. Approximate 100 anchorage independent individual clones were isolated and cultured. The genomic DNA of a single colony was extracted and PCR was conducted to amplify the hairpin DNA of the integrated shRNAs and followed by submitting to Sanger DNA sequencing. Though most PCR products failed to produce informative DNA sequencing results because of either technical issues or multiple species of shRNAs existing in a single genome, we still were able to identify a total of 6 individual genes targeted by 6 unique shRNAs through sequencing the proviral shRNAs from these individual clones (Table 1 and S1 Table for detailed information). Among the 6 candidate transformation suppressors, INPP4B is a known tumor suppressor involving in breast and prostate cancer [22,23], the roles of the rest candidate genes in suppression of cell transformation have not been clear or well- characterized yet. Since the Sesn2 gene has been implicated in cellular regulation via p53 signaling pathway, thus, we chose Sesn2 as the top candidate tumor suppressor and validated its cellular functions both *in vitro* and *in vivo*.

**Table 1:** Candidates of malignant transformation suppressors identified from an shRNA based genetic screen in BEAS-2B cells.

Target gene symbol	Description	Validation
INPP4B *	Inositol polyphosphate 4- phosphatase	Yes
Sesn2 *	Homo sapiens sestrin 2	Yes
TIAR**	T-cell intracellular antigen 1-related protein	Yes
ACRC**	Acidic repeat containing	N/A
NUP210 **	Nucleoporin 210kDa	N/A
LMTK3 **	Lemur tyrosine kinase 3	N/A

* An identical shRNA against INPP4B or Sesn2 transcript was identified three times

from three independent transformed colonies.

** These unique shRNAs were identified only one time from only one transformed colony

### Sesn2 suppresses malignant transformation of BEAS-2B cells

**Fig 2:**
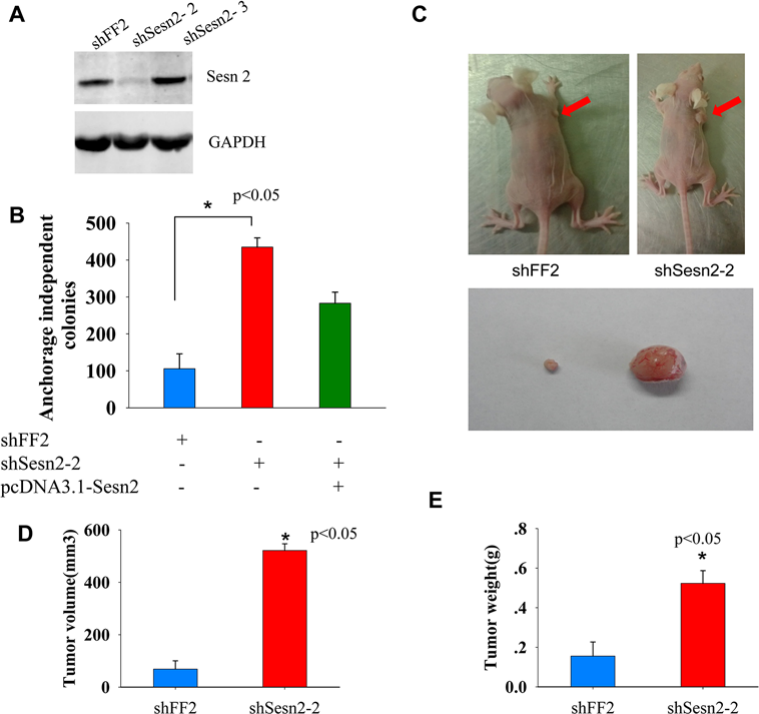
Sesn2 inhibits *in vitro* cellular transformation and *in vivo* tumor formation of lung epithelial cells. **(A)** Knocked down of endogenous Sesn2 by two independent shRNAs targeting 3’ UTR of Sesn2 transcripts, was confirmed by immuno-blotting. **(B)** The average number of colonies formed in shSesn2-2 transformed cells were 4 times higher than shFF2 transformed cells, *p<0.05. Ectopic expression of Sesn2 re-suppressed the anchorage independent growth in the shSesn2-2 expressing cells. **(C)**, **(D)** and **(E)** Knockdown of Sesn2 in BEAS-2B cells led to enhanced tumor formation in nude mice. The Sesn2 silenced cells were transplanted into the armpits of *Balb/c* nude mice, xenografts were formed one month later post transplantation and photographed, red arrows indicated formed tumors under the mouse skin (upper panel of C). The image of two representative dissected tumors was shown in the lower panel of C. The average tumor volumes and weights from transplanted mice with Sesn2 silencing and FF2 control cells were measured and plotted in D and E, data are presented as mean ± standard deviation per mouse group, *p<0.05 by the Student t-test.

Sesn2, first identified as Hi95, is a hypoxia inducible protein and displays a function of inhibiting mTOR signaling pathway [18]. Some researches showed that it may act as a tumor suppressor because of its inhibition of cell proliferation, but there is no direct evidence to prove that [16]. From our shRNA based genetic screen and DNA sequencing results, we found three BEAS-2B transformed colonies harboring an identical shRNA targeting the 3’ UTR of Sesn2 transcripts (designated as shSesn2-1, for shRNA sequence information see S1 Table). To rule out off-target effects, we designed and tested two more shRNAs (named as shSesn2-2 and shSesn2-3, see S1 Table) targeting the different regions of the 3’ UTR of Sesn2 transcripts. The cell lines expressing Sesn2 targeting shRNAs were analyzed by Western blotting and the level of endogenous Sesn2 has been reduced by about 80% in the shSesn2-2 expressing cell line (Fig 2A). We seeded those Sesn2-2 silencing cells in soft agar plates. After 3 weeks of growth, the shSesn2-2 silencing cell exhibited strong colony formation ability with 435±25 colonies per 10cm plate, while fewer and smaller colonies (106±40 per plate) were formed in the shFF2 control cell seeded plates (Fig 2B). Furthermore, ectopic expression Sesn2 driven by pcDNA3.1 vector in the shSesn2-2 silencing cell partially re-suppressed the colony formation ability elicited by shSesn2-2 (Fig 2B), indicating the observed phenotypes were likely attributable to the reduction of Sesn2, not caused by shRNA off-target effects. To test the *in vivo* tumor restraining ability conferred by Sesn2, the shSesn2-2 and shFF2 cells were injected subcutaneously into the right ampits of the Balb/c nude mice. At the 28th day following implantation, the formed xenograft was dissected from each mouse, the volume and weight of each tumor were measured and compared. As shown in Fig 2C, a strong xenograft tumor formation ability was observed in shSesn2-2 expressing cells (for the summary of all tumors formed by Sesn2 knockdown cell line in two batches of nude mice, see S2 Fig). The average xenograft tumor volumes in shSesn2-2 and shFF2 transplanted mice are 521.45 ± 25.51mm^3^and 69.02 ± 31.48mm ^3^ (Fig 2D), and the average tumor weights were 0.523±0.064 g and 0.156±0.071g respectively (Fig 2E), these differences are statistically significant (p< 0.05 by the Student’s t-test). Taken together, our results indicate that knockdown of Sesn2 promotes malignant transformation of BEAS-2B cells both *in vitro* and *in vivo*, and Sesn2 may act as a potential tumor suppressor in lung epithelial cells.

### Silencing Sesn2 by shRNA promotes both cell growth and cell migration

**Fig 3:**
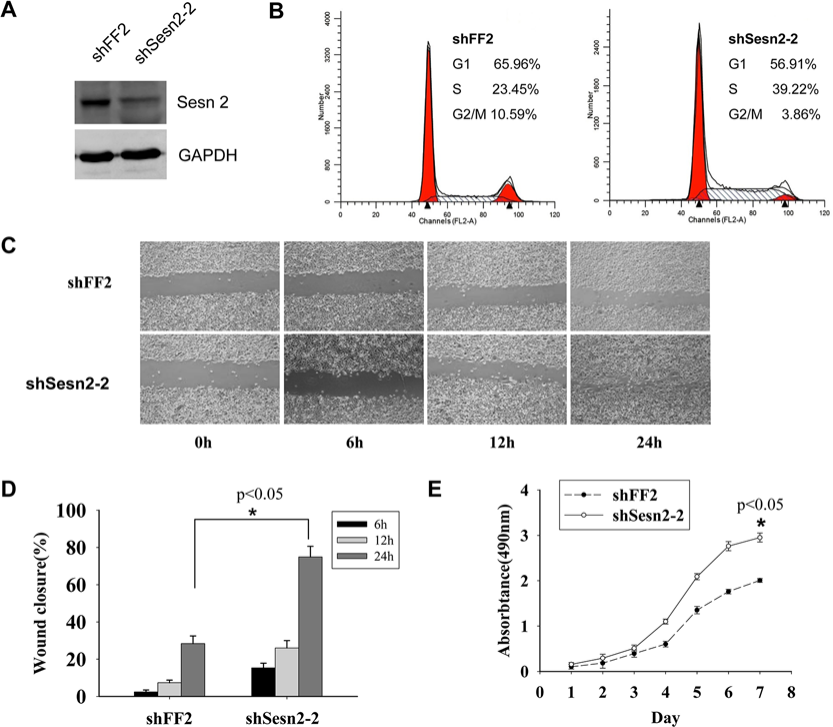
Silence of Sesn2 promotes cell growth and cell migration. **(A)** Conformation of Sesn2 knockdown in A549 cells by immuno-blotting. **(B)** The cell cycle profiles of lung adenocarcinoma A549cell lines expressing shSesn2-2 and shFF2. Cells were stained with propidium iodide and analyzed by flow cytometry. The higher S phage proportion detected in shSesn2-2 cells suggested faster cell cycle progression compared to shFF2 cells. **(C)** Representative images of wound healing experiment performed in shSesn2-2 and shFF2 expressing cells at the indicated times. The average wound closure percentages in each cell group were plotted in **(D)**, significant healing difference were observed after 24 hour of wound healing, *p<0.05. Experiments were performed in triplicate. **(E)** The cell growth rates of shSesn2-2 and shFF2 expressing cells were determined by MTT assays and compared by the two-way Anova test (*p<0.05).

Accelerated cell growth and cell migration are the characteristics of malignant transformed cells. To explore the effects of Sesn2 on cell growth and cell migration, we established a Sesn2 silencing cell line by infecting retroviral shSesn2-2 into lung adenocarcinoma A549 cells (Fig 3A). Cell cycle profiles in the resulting cell lines were determined by flow cytometry. A high S phase proportion was detected in the shSesn2-2 cells than that in shFF2 cells (39.22% vs 23.45%, Fig 3B) indicating silencing of Sesn2 caused accelerated cell cycle progression. Then, we monitored cell growth by MTT assay, and found the shSesn2-2 cells proliferate faster than shFF2 cells (p<0.05, Fig 3E). Wound healing experiments were performed in shSesn2-2 and shFF2 cells to check the relation between the expression of Sesn2 and cell migration. Prior to the assay, shSesn2-2 and shFF2 cells were pre-treated with mitomycin C to ensure that the closure of the wound was due to cell migration but not cell proliferation. The average closure areas were 15.30±2.56, 26.03±3.96, 74.93±5.72 at 6h, 12h and 24h following scratch creation in shSesn2-2 cells and 2.42±1.05, 7.37±1.38, 28.4±4.08 in shFF2 cells respectively (Fig 3C and Fig 3D). The rapid wound closure in shSesn2-2 cell supported that deficiency of Sesn2 promotes cell migration of lung adenocarcinoma A549 cells.

### Sesn2 suppresses Akt-mTOR-p70S60K signaling

**Fig 4:**
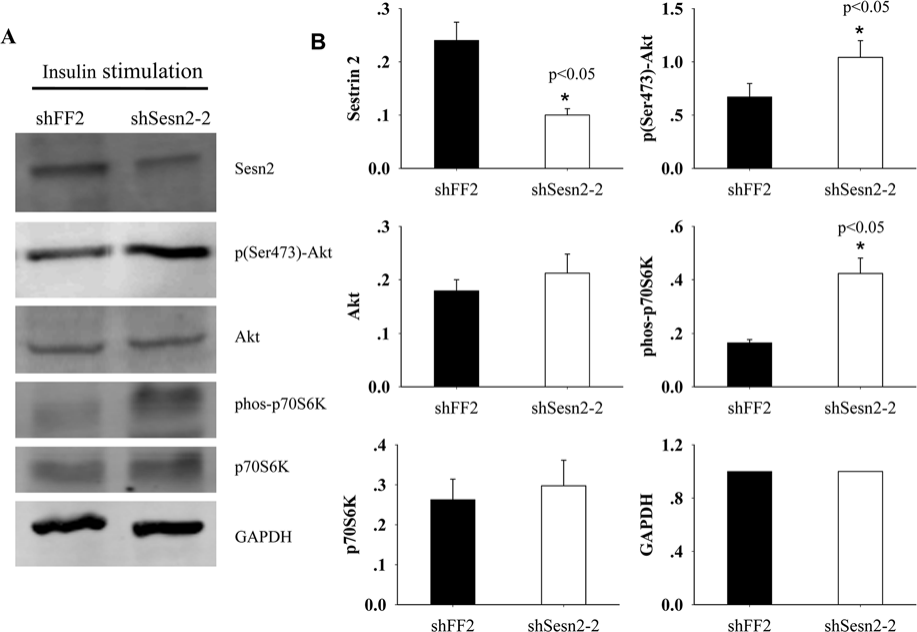
Down-regulation of Sesn2 by shRNA causes enhanced activation of Akt-mTOR-p70S6K signaling upon insulin stimulation. (A) A549 cells stably expressing shSesn2-2 or shFF2 were serum-starved overnight and re-stimulated with 100nM insulin for 20 min, the expression levels of Akt, p70S6K and their phosphorylated species were detected by immuno-blotting with indicated antibodies. (B) The intensity of protein bands on the immuno-blots was quantified with densitometry scanning. The relative expression level of each protein was normalized based on the level of GAPDH in each lysate and plotted in B, data were shown as mean ± standard deviation (SD) from three independent experiments. *p<0.05 indicates a significant difference in the expression levels of the examined proteins in the two cell lines.

The implication that Sesn2 involves in regulating the transformed state of BEAS-2B epithelial cells prompts us to determine which molecular circuit might be affected by the loss of Sesn2 function. As Akt-mTOR signal pathway plays an important role in various tumors, and the Sestrin family has been implicated in the suppression of mTORC1 activation by binding to AMPK [24], we checked the effects on mTOR activation upon silencing of Sesn2 in A549 cells. We detected the levels of phosphorylated p70S6K, a downstream target of mTOR and an indicator for mTOR activation, and the phosphorylated Akt at Ser473, an active species of Akt, in A549 cells expressing shSesn2-2 or shFF2. Under normal cell culture condition, the basal levels of phosphorylated p70S6K and phosphorylated Akt in both cell lines were barely detectable (data not shown). However, after addition of 100 nM insulin into the serum starved two cell lines for 20 min, phosphorylated p70S6K was detectable in both cell lines, but much more phosphorylated p70S6K was observed in Sesn2 knockdown cells (Fig 4A). These data indicate mTOR-p70S6K pathway is more activated upon Sesn2 silencing by shRNAs. Several previous studies reported insulin induced Akt activation in mice requires intact mouse Sesn2 [25], but we still observed significant activation of Akt upon insulin stimulation in Sesn2 silencing cells. Our observation may reflect the controversial role of Sesn2 in response to different nature of stress. In agreement with our findings, the over-expression of Sesn2 caused less phosphorylated Akt-S473, renders MCF-7 cells more sensitive to ionizing radiation [17]. Collectively, our findings indicated that Sesn2 may act as a tumor suppressor gene that can inhibit cancer cell proliferation viability through the regulation of Akt-mTOR-p70S6K signal pathway.

### Low expression levels of Sesn2 correlate with poor survival in Chinese lung cancer patients

In order to establish a causal relationship between disruption of Sesn2 function and lung tumor genesis, we collected biopsy specimens from 77 lung cancer patients diagnosed by the tumor hospital of Harbin Medical University (the detailed clincopathological features of 77 Chinese lung cancer patients were summarized in S2 Table). The genomic DNA was extracted from those lung cancer tissues and their adjacent normal lung tissues. We amplified the 3rd and 4th exons (which are the two DNA elements for encoding the function domain of Sesn2) of Sesn2 genes and subjected the PCR products to Sanger DNA sequencing. Of all the sequenced samples, only two point mutations Val (GTA)76 to Ala (GCA), Thr (ACG)103 to Lys (AAG) in the exon 3 of Sesn2 gene were detected (see Table 2) and this two mutations occurred simultaneously. Moreover, only one lung cancer patient (1 out of 77) was found harboring the two mutations. The very low mutation rate of Sesn2 gene in human lung cancer suggests the structural alteration of Sesn2 gene is not a major cause contributed to the malignant transformation of lung epithelial cells. To test whether this Sesn2 mutant interferes with the function of endogenous wild type Sesn2, we introduced an Sesn2 cDNA harboring the Val76 to Ala and Thr103 to Lys mutations by pcDNA3.1 expression vector into the BEAS-2B cells. The transformed cells with over-expression of the Sesn2 mutant failed to elicit obvious anchorage independent growth compared to cells transformed with the empty vector (p> 0.05) (S2 Fig). This observation suggested the mutations of Val76 to Ala and Thr103 to Lys in the exon 3 of Sesn2 gene may not be able to produce a strong dominant negative activity at least in BEAS-2B cells.

**Table 2:** Mutations of Sesn2 gene identified from 77 Chinese lung cancer patients.

	Exon 3			Exon 4		
	mutation Val76 to Ala Thr103 to Lys	no mutation	p value	mutation	no mutation	p value
High expression of Sesn2	0	20	>0.05	0	20	>0.05
Medium & low expression of Sesn2	1	56		0	57	

**Fig 5:**
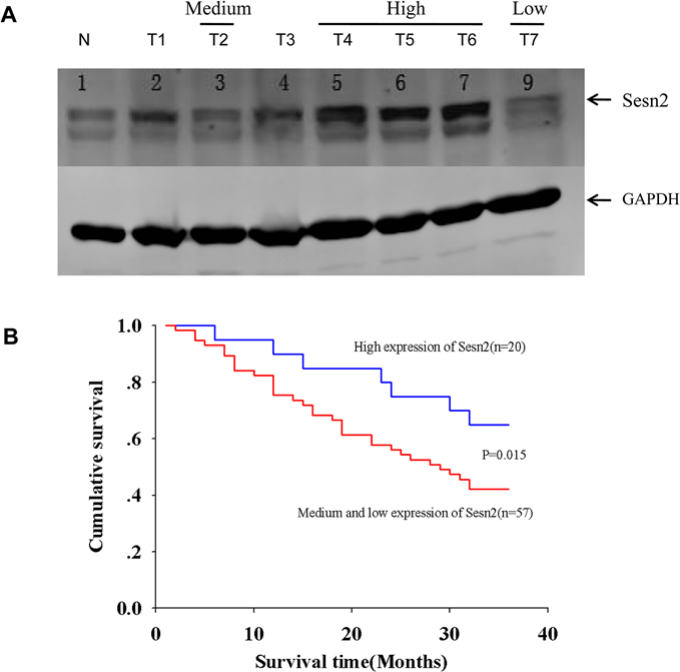
Low levels of Sesn2 expression associate with poor survival in Chinese lung cancer patients. **(A)** Tissue homogenates prepared from resected lung cancer tissues were subjected to immuno-blotting probed with the indicated antibodies. Shown is a representative image of a Western blot. Capital letter N depicts a normal tissue adjacent to a cancer tissue, and capital letter T with a number indicates a cancer tissue sample from an individual lung cancer patient. Sesn2 expression level was quantified by densitometry scanning and normalized based on the signal of loading control GAPDH, detailed methods for defining the protein expression level were described in material and method section. **(B)** Patients were divided into the high expression and the combined medium & low groups based on their expression levels of Sesn2 examined in A. Kaplan Meier survival analysis showed the low expression of Sesn2 positively correlated with a poor survival rate of the examined lung cancer patients during a three-year follow up upon their diagnosis, p = 0.015 by a log-rank test indicates a significant difference of survival rate between the two patient groups.

Next, we determined the protein levels of Sesn2 in those lung cancer tissues by immuno-blotting. Total tissue lysates were generated by homogenization and applied to SDS-PAGE resolution. The protein level of Sesn2 was revealed by Western blotting with antibodies against Sesn2, signal intensity of Sesn2 protein was quantified by densitometry scanning (Fig 5A). We define the relative expression of Sesn2 as low, medium and high in comparison with Sesn2 level in their adjacent normal lung tissues (the details for this definition were described in material and method section). In summary, of the 77 lung cancer patients examined, 57 patients were scored with low and medium expression of Sesn2, 20 patients were scored with high expression of Sesn2. We analyzed the expression distribution of Sesn2 in respect to their clinicopathological categories such as squamous-cell carcinoma, adenocarcinoma and small cell carcinoma as well as sex, age and the grade of cell differentiation, we found there is no significant difference of Sesn2 expression under those categories (details see S2 Table). Thus, Sesn2 expression levels are not likely affected by these clinicopathological features of lung cancer patients.

We divided the examined patients into the high expression and the combined medium & low groups based on their expression levels of Sesn2. A three-year survival follow up of those 77 lung cancer patients was carried out upon their diagnosis, the Kaplan Meier analysis indicated the three-year survival rates in the high and the low & medium groups were 65.00% and 42.11% respectively, log-rank test indicated a significant decrease in the 3-year survival rate of the low & medium group (p<0.05) compared to the high group (Fig 5B). Therefore, we conclude the low expression level of Sesn2 associates with a poor survival rate in Chinese lung cancer patients, and the expression level of Sesn2 may serve as a potential prognostic marker for lung cancer patients in the clinic.

## Discussion

A loss-of-function screen by using shRNA library is a proven powerful approach for identification of new and unanticipated tumor suppressors in an unbiased manner. In this study, we applied this approach to identify genes suppressing oncogenic transformation in human lung epithelial cells. We have identified several genes implicated in cancer pathogenesis. Although the genes isolated via this strategy may not be frequent targets of mutation in tumors, they may nonetheless reveal novel pathways relevant to tumorigenesis. For example, we isolated inositol polyphosphate 4-phosphatase type II(INPP4B)gene, a known tumor suppressor in breast, prostate, and among other malignancies [22,26]. The mutation or low expression of INPP4B has been closely related to the poor prognosis of these cancers [23]. In addition, identification of T-cell intracellular antigen 1-related protein (TIAR) further supports previous findings that TIAR is down-regulated in a subset of epithelial tumors and HeLa cells with reduced TIAR by shRNA produced larger epithelial xenografts in nude mice [27]. These known cancer-relevant genes identified through our screens suggested other candidate genes might be more or less related to restraining anchorage-independent proliferation, further experiments are in need to determine the extent to which these genes are involved in lung tumorigenesis.

AKT-mTOR pathway has played a critical role in cancer development by regulating cell proliferation, apoptosis and angiogenesis [28]. We showed that lack of Sens2 function enhances the activity of AKT-mTOR signaling may be an important mechanism underlying the ability of Sesn2 to restrain the transformed state. In agreement with our finding that Sesn2 is a cellular transformation suppressor in lung epithelial cell BEAS-2B, Sestrin family has been implicated in cellular proliferation and cell survival via suppressing the mammalian target of rapamycin complex 1 (mTORC1) activity by the activation of AMP-dependent protein kinase (AMPK) in a p53 dependent manner [15,16,29]. Furthermore, Sestrins mediate the DNA damage-sensing pathway composed of ataxia telangiectasia mutated (ATM) and p53, even though the underlying mechanism is unclear so far [16]. Interestingly, the role of Sesn2 in regulating cell survival remains controversial. For example, upon DNA damage induced oncogenic transformation, elevated Sesn2 in MCF-7 cells promotes IR induced apoptotic cell death [17]. However, under the energetic stress such as ATP deficiency caused by 2-deoxyglucose (2-DG) exposure, Sesn2 protected 2-DG induced cell death in both LNCaP cells and mouse embryonic fibroblasts [18].

Most strikingly, the Sesn2 knockout mouse presented no tumor formation, even no obvious shorted lifespan which is in contrast to its roles in tumor suppression and antiaging demonstrated previously [25]. Why there is such a phenotypic discrepancy occurred between the *in vitro* cellular transformation and the *in vivo* mouse model? Given the fact there are three Sentrin members in mammals with high homology (e.g. the homology between Sesn1B and Sesn2 is 71%), most likely, Sesn2 may be play a redundant role in mice and its deficiency may be compensated by its close homologue such as Sesn1B or Sesn3. However, on a cellular level, execution of such compensation maybe dependent on the genetic background under a given cell line, which may completely differ from the situation on a whole organism such as certain strains of mice.

In contrast to previous finding that insulin induced activation of Akt requires presence of Sesn2 demonstrated by using the Sesn2 knock out mouse model [25], our data suggested insulin treatment was still able to stimulate the enhanced phoshorylation of Akt-S473 in A549 cells with Sesn2 knocked down by shRNAs. Given the fact that AKT cascade can be regulated by many membrane proteins including receptor tyrosine kinases, integrins, G protein-coupled receptors [30], it is likely that the activation of Akt in human cells is partially independent of the intact Sesn2. In support of our findings, the over-expression of Sesn2 caused less phosphorylation on Akt-pS473 in MCF-7 cells [17], and the knockdown of AMPKα, a functional partner of Sestrins for inhibition of mTORC1, did not influence the increase of Akt phosphorylation stimulated by FoxO1activation [29].

Genetic alterations often contribute to the inactivation of tumor suppressor genes. Loss of heterozygocity (LOH) in Sesn1 (6q21) and Sesn2 (1p35) is also often observed in diverse human cancers [14,31]. Unlike its master p53 gene, we found the mutation rate of Sesn2 gene in lung cancer tissue is very low. Among tissue specimens resected from 77 lung cancer patients, only one was detected with point mutations in Sesn2 gene. Given the fact that 60–80% of lung cancer tissues harbor p53 mutations, and Sesn1 and Sesn2 are direct targets of p53 mediated transcription, the role of Sesns in tumor genesis may be part of a mechanism underlying the impairment of p53 in lung cancer cells.

The clinical relevance of Sesn2 expression level was evaluated in lung cancer tissue specimens resected from 77 lung cancer patients. Though the Sesn2 protein levels vary a lot in the examined lung tissues, there is a clear positive correlation between the low Sesn2 expression level and a poor survival rate in the examined lung cancer patients. Surprisingly, in contrast to most tumor suppressors generally down-regulated in cancer tissues, we have observed a significant portion of high expression of Sesn2 (20 out of 77) in the examined lung cancer patients, their Sesn2 levels even are much higher than their adjacent normal lung tissues. Initially, Sestrins have been implicated in response to oxidative stress, inactivation of Sestrins and accumulation of reactive oxygen species (ROS) have been connected to carcinogenesis [32]. Under oncogenic conditions such as chronic inflammation, prolonged growth factor signaling and high levels of oxidative stress maybe detrimental to the survival and propagation of cancer cells, as a result of antioxidant mechanisms, it is not surprised to observe up-regulated Sestrins in certain parts of lung cancer tissues [13].

Despite that we have identified a number of candidate suppressors for cellular transformation in human lung epithelial cells, it is obvious that this screen is far from saturation. First at all, we only screened 6 pools out of the total 72 pools of the utilized shRNA library due to time and labor limitations. Secondly, technical drawbacks such as loss of shRNA representations during library plasmid preparation and transformation into packaging cells hindered the efficiency of our screen. Thirdly, the current library may lack of sufficiently penetrant shRNAs to elicit the expected transformation phenotypes. Lastly, the mechanisms by which tumor suppressor genes are inhibited may vary among different tumor cells [8]. Therefore, there remains a significant potential for shRNA based screens in the future discovery of tumor suppressor genes in human lung epithelial cells, and continuing screening for new tumor suppressor will shed more light into the molecular mechanism driving the malignant transformation of lung cells and provide more novel therapeutic targets for lung cancer prevention and treatment in the future.

## Materials and Methods

### Ethics statement

Lung tumor specimens were obtained from lung cancer patients who underwent surgical operation for lung cancer at the tumor hospital, Harbin Medical University (Harbin, China). Written consent was obtained from all involved patients with procedures that were approved by the Heilongjiang Province Ethics Committee (Harbin, China). All animal protocols were approved by the Animal Ethics Committee of the Harbin Medical University in accordance with the national regulations of China.

### Vector and retrovirus packaging and shRNA screen in soft agar

The retroviral shRNA library constructed in pSM2 vector has previously been described [10], VSV-G expression plasmid and MSCV-PM FF2 plasmid were a kind gift from Dr. Stephen J. Elledge of Harvard Medical School. The retrovirus was packaged using GP2-293 packaging cell line. The retroviral supernatants were generated by transient transfection of GP2-293 cell with shRNA plasmids and pVSV-G expression plasmid as described in reference [21]. The virus collected at 48h and 72h after transfection. BEAS-2B cells were infected with a retroviral shRNA library with multiplicity of infection (MOI) at 1.0. Infected cells were selected with puromycin for 1 week and used for the subsequent soft agar assays. Anchorage-independent assays were performed as described previously [11]. Briefly, control and shRNA library infected BEAS-2B cells were plated at 10,000 cells/ per 10cm plate with culture medium containing 0.35% low melting agarose in the top layer and 0.6% low melting agarose in the base layer. The plates were incubated at 37°C and 5% CO2 for 3 weeks. The colonies were stained with crystal violet (Sigma), counted and photographed. For each assay, three independent experiments were performed. Individual anchorage-independent colonies were isolate and expanded for genomic DNA isolation and PCR amplification. PCR amplification of the half hairpin was done with the primer pairs, forward: 5’-TAATCTCGAGTAGTGAAGCCACAGATGTA-3’;

reverse: 5’- CGGACGCGTGAAAAAAGTGATTTAATTTATACCATT-3’. The PCR products were subjected for Sanger DNA sequence and analyzed by Blast in NCBI for identification of its target gene.

### Cell culture

Cell lines used were obtained from the American Type Culture Collection (Manassas, VA, USA) The human bronchial epithelial BEAS-2B cells were cultured in Bronchial epithelial cell grown medium (BEGM, Lonza). Retrovirus packaging cell line GP2-293 was maintained in DMEM (Gibco) supplemented with 10% fetal bovine serum (PAA), 100 mg/ml penicillin and 100 mg/ml streptomycin (Gibco Life Technologies). Human lung adenocarcinoma epithelial cell line A549 was cultured in F12K (Gibco) containing 10% fetal bovine serum (PAA), 100 mg/ml penicillin and 100 mg/ml streptomycin (Gibco Life Technologies). All cell lines were incubated at 37°C with 5% CO2. Cell lines stably expressing shRNA library or desired shRNAs were generated by infection with the indicated retroviral supernatants in the presence of 8μg/mL polybrene for 48h and selected with 0.6μg/mL puromycin for 1 week.

### Antibodies and chemical reagents

Antibodies against Sesn2, Akt, phospho-Akt-S473, p70S6K, phosphorylated p70S6K, GAPDH, etc, were purchased from Santa Cruz and/or Cell signaling technology. All chemicals reagents including insulin, mitomycin C, antibiotics were purchased from Sigma-Aldrich.

### Cloning, mutagenesis and plasmid construction

Total RNA was extracted from BEAS-2B cells using Trizol reagent (Invitrogen). Complementary DNA was synthesized by the Transcript Reverse Transcriptase kit (Invitrogen). For stable expression of 3xFlag-Sesn2, the coding sequences of Sesn2 were amplified from above cDNAs by following primer pairs:

Sesn2-F: 5’-TACACTCGAGATGATCGTGGCGGACTCCGAGTGC-3’

Sesn2-R: 5’-GCTAGGTACCTCAGGTCATGTAGCGGGTGA-3’

and cloned into pcDNA3.1(-) 3xFlag through XhoI/KpnI restriction sites.

To construct pcDNA3.1(-) 3xFlag-Sesn2 mutant harboring Val76 to Ala and Thr103 to Lys, site-directed mutagenesis was performed by using high fidelity Pyrobest DNA polymerase (TaKaRa) and following mutagenesis with primer pairs: Mu76F1:5’-ATGTCCTCTGGGCGAGCAGACAACCTGGCAGTG-3’

Mu76R1:5’-CACTGCCAGGTTGTCTGCTCGCCCAGAGGACAT-3’

Mu103F2: 5’-TACCTGCTGCTGCACAAGGATGGTCCCTTGGCC-3’ Mu103R2: 5’-GGCCAAGGGACCATCCTTGTGCAGCAGCAGGTA-3’, amplified plasmids were digested by DpnI, followed by transformation into E. coli, WT and mutant Sesn2 genes were verified by DNA sequencing. For generating retroviral pSM2 vectors expressing shSesn2-2 and shSesn2-3, two primers (miR30-XhoI-F: 5-CAGAAGGCTCGAGAAGGTATATGCTGTTGACAGTGAGCG-3’, miR30-EcoRI-R: 5’-CTAAAGTAGCCCCTTGAATTCCGAGGCAGTAGGCA-3’) were used for generating desired shRNA cassettes by PCR and resulting amplicons were inserted into pSM2 through XhoI /EcoRI restriction sites. The detailed hairpin DNA sequences targeting 3’ UTR of Sesn2 transcript were listed in S1 Table. The desired cells were transfected by Sesn2 exprerssion plasmid with lipofectamine 2000 (Invitrogen) or infected with retroviruses expressing shSesn2 and stable transfectants were selected in the presence of 200 ug/ml hygromycin B or 2.0 ug/ml puromycin (Sigma-Aldrich). Protein expression levels in these resulting clones were determined by Western blotting with specific antibodies against the proteins.

### Mouse xenograft tumor model

BEAS-2B cells (1×10^7^ cells in 0.2 ml phosphate-buffered saline) expressing shSesn2-2 or shFF2 were injected subcutaneously into the right flanks of 6-week-old male Balb/c nude mice (Charles River laboratory), separately. Each cell line was injected into 6 mice for tumor formation assays. Tumor volume (mm^3^) was measured as following formula: volume = (shortest diameter)^2^ x (longest diameter) x 1/2. All experimental procedures were in accordance with the Animal Ethics Committee of the Harbin Medical University. One month post implantation, the mice were sacrificed by cervical dislocation and tumor xenografts were dissected and fixed in 4% paraformaldehyde. Tumors were weighed, measured and photographed.

### Cell proliferation assay

Cell proliferation was performed by MTT assay (Promega, USA) according to the manufacturer’s protocol. Briefly, 1,000 cells were seeded in a 96-well plate and cultured for the next day. 20μL of MTT solution was added into each well of a 96-well plate containing the cells in 100μL of culture medium. Incubate the plate at 37°C for 4 hours in a humidified, 5% CO2 atmosphere. The absorbance was quantified by a 96-well plate reader at the wave-length of 490 nm. Cells were measured on seven consecutive days. The experiment was repeated three times independently.

### Cell migration assay

Cell migration assay was performed by measuring the wound healing area of the control and Sesn2 knockdown cells. Cells were seeded into a six-well plate and allowed to reach 100% confluence. Cells were then treated with mitomycin C (final concentration at 50ug/ml, Sigma) to inhibit cell proliferation for 2 hours before scratches were generated by a 100μL tip. Cells were washed by PBS to remove all the dislodged cells. The medium was replaced with serum free medium. The cells were photographed after 0 h, 12 h and 24 h. Migration of the cells was measured by determining the wound healing areas of the control and Sesn2 knockdown cells.

### Cell cycle assay

Sesn2 knockdown or control cells were harvested from a 10cm plate and fixed with 70% ethanol at 4°C overnight. 1ml of PBS buffer containing RNase A (100ug/ml) and propidium iodide (40ug/ml) was added to each cell sample and the cell cycle profile was analyzed by a flow cytometer (BD Biosciences).

### Human lung cancer specimen preparation and Western blotting

Lung tumor specimens (0.25–1.0 g per sample) were obtained from 77 lung cancer patients who underwent surgical operation for lung cancer at the tumor hospital, Harbin Medical University from Jan, 2010 to Sep. 2010. Tissue specimens were snap-frozen in liquid nitrogen and stored at -80°C for later use. Patients did not receive any treatment before surgery, and signed informed consent forms for tissue collection. The pathological stage, grade, and nodal status were obtained from the primary pathology reports according to TNM staging. After surgical removal and prevention chemotherapy, these patients were followed up to a total of 36 months since the time of their diagnosis. Lysates of both tumor tissues and their adjacent normal lung tissues were prepared by homogenization in a nitrogen liquid bath. The homogenate was clarified by 14,000 rpm centrifugation for 30 minutes at 4 degree, protein concentration was determined by the Bradford assay, 60 ug of each supernatant was resolved on a 10% SDS-PAGE. The proteins were transferred to PVDF membrane and incubated with specific primary antibodies and appropriate secondary antibodies, and visualized with the Odyssey imaging system (Li-COR). The intensity of bands of Western blot was quantified by densitometry scanning (Li-COR). After normalization with GAPDH in each tissue, we compared the signal of Sesn2 in each cancer tissue versus the signal of Sesn2 in each adjacent normal lung tissue, the ratio in each patient with equal or greater than 80% was scored as high expression of Sesn2, in between 80% and 40% was scored as medium expression of Sesn2 and lower than 40% as low expression of Sesn2. We divided these patients into the high expression and medium & low expression groups to monitor and compare the overall median survival rates between the two groups.

### Statistical analysis

Data were expressed as mean ± standard deviation (SD). The difference in cell growth rates between the two cell lines was determined by the two-way ANOVA analysis. Comparison of colony numbers, tumor weights and volumes and relations between patient clinicopathological features and Sesn2 expression levels were determined by a Student’s T test or Chi-Square test. Overall survival in relation to Sesn2 expression status was evaluated by the Kaplan—Meier survival curve and the log-rank test. All analyses were performed using SPSS for Windows 11.0.1 software (SPSS Inc., Chicago, IL, USA). p< 0.05 was considered statistically significant.

## Supporting Information

S1 Fig Knockdown of Sesn2 enhanced xenograft tumor formation in nude mice. All tumors were removed from two different batches of implantation experiments, and tumors were fixed in 4% paraformaldehyde for later use.
(TIF)

S2 Fig Sesn2 mutant (Val 76 to Ala, Thr 103 to Lys) exhibited no obvious dominant negative property. BEAS-2B cells were transfected with pcDNA3. 1-Flag-Sesn2 mutant or empty vector and stably transformed cells were seeded into soft agar medium for measuring anchorage independent growth for three weeks. Colonies were stained by crystal violet and average numbers of colony from three independent experiments were plotted. Ectopic expression of Sesn2 mutant (Val 76 to Ala, Thr 103 to Lys) in BEAS-2B cell lines were confirmed by Western blotting probed with anti-Flag antibody, the GAPDH signal was served as a loading control.
(TIF)

S1 Table Sequences of shRNAs identified and used in this screen. (DOC)

S2 Table Clinicopathological features of 77 Chinese lung cancer patients and their relation to Sesn2 protein expression levels. (DOC)
