# Supplementary figures and images for: An ShRNA Based Genetic Screen Identified Sesn2 as a Potential Tumor Suppressor in Lung Cancer via Suppression of Akt-mTOR-p70S6K Signaling
Source: PLoS One. 2015 May 11;10(5):e0124033. doi: 10.1371/journal.pone.0124033 (PMC4427398; doi:10.1371/journal.pone.0124033)

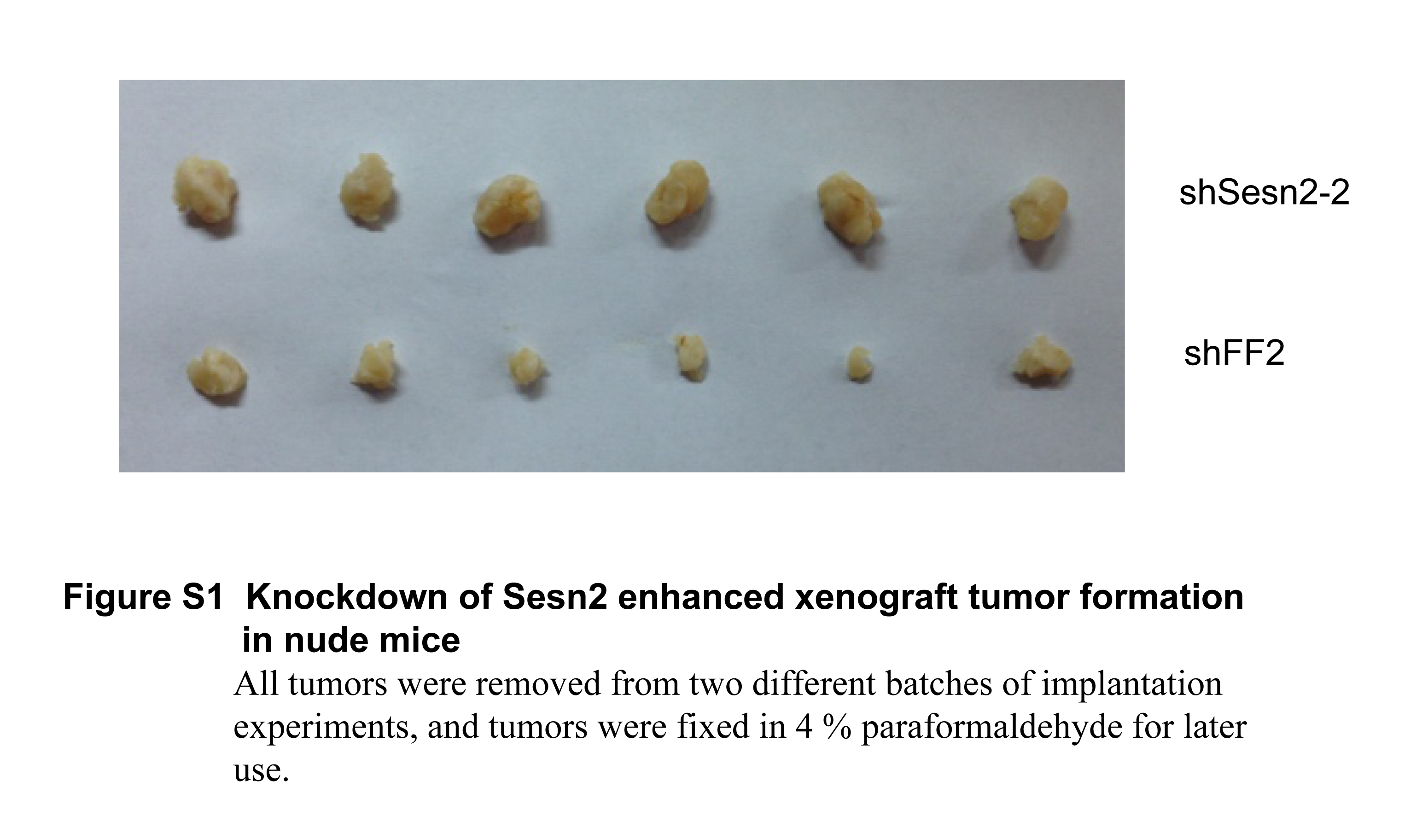

Supplement: S1 Fig — All tumors were removed from two different batches of implantation experiments, and tumors were fixed in 4% paraformaldehyde for later use. (TIF) [file pone.0124033.s001.tif]

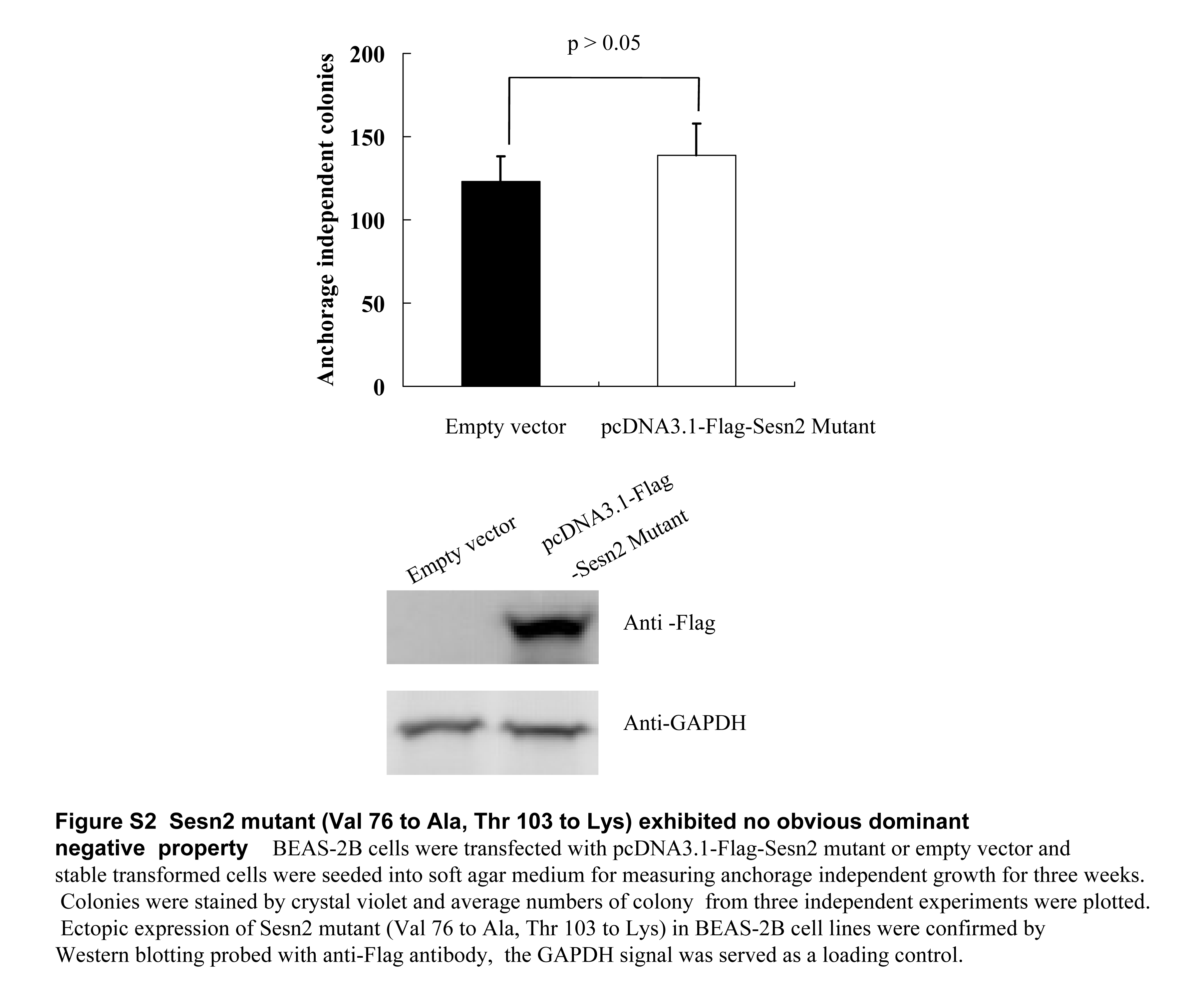

Supplement: S2 Fig — BEAS-2B cells were transfected with pcDNA3. 1-Flag-Sesn2 mutant or empty vector and stably transformed cells were seeded into soft agar medium for measuring anchorage independent growth for three weeks. Colonies were stained by crystal violet and average numbers of colony from three independent experiments were plotted. Ectopic expression of Sesn2 mutant (Val 76 to Ala, Thr 103 to Lys) in BEAS-2B cell lines were confirmed by Western blotting probed with anti-Flag antibody, the GAPDH signal was served as a loading control. (TIF) [file pone.0124033.s002.tif]
