# Supplementary material for: An ShRNA Based Genetic Screen Identified Sesn2 as a Potential Tumor Suppressor in Lung Cancer via Suppression of Akt-mTOR-p70S6K Signaling
Source: PLoS One. 2015 May 11;10(5):e0124033. doi: 10.1371/journal.pone.0124033 (PMC4427398; doi:10.1371/journal.pone.0124033)
Supplement: S1 Table — (DOC) [file pone.0124033.s003.doc]

**S1 Table Sequences of shRNAs identified and used in this screen**

| shRNA name | shRNA antisense sequence |
| --- | --- |
| sh-INPP4B* | TTACGATCACGGACAGGAGCCA |
| sh-Sesn2-1* | TTATAATCTAATACTCCCTCCT |
| sh-Sesn2-2** | TAATGTGGCACACTGGCCAGGC |
| sh-Sesn2-3** | ATTAATTCCGAGCTTGGCCCGG |
| sh-TIAR* | TTCCATAATTTGTCCAAATGGT |
| sh-ACRC* | ATACTTCTGCACATACACAGCA |
| sh-NUP210* | TTATTGTGTACTGATAGGCGCC |
| sh-LMTK3* | TAATCAGCAGAAACGGCAGCGT |

* These shRNAs were identified by DNA sequencing from transformed colonies on soft agar

** The two shRNAs were generated by ourselves and cloned into pSM2 vector.
