# Supplementary material for: An ShRNA Based Genetic Screen Identified Sesn2 as a Potential Tumor Suppressor in Lung Cancer via Suppression of Akt-mTOR-p70S6K Signaling
Source: PLoS One. 2015 May 11;10(5):e0124033. doi: 10.1371/journal.pone.0124033 (PMC4427398; doi:10.1371/journal.pone.0124033)
Supplement: S2 Table — (DOC) [file pone.0124033.s004.doc]

**S2 Table Clinicopathological features of 77 Chinese lung cancer**

**patients and their relation to Sesn2 protein expression levels**

|  | Sesn2 | |  |
| --- | --- | --- | --- |
|  | High expression | Medium and low expression | p value |
| Sex |  |  | 0.118 |
| Male | 12 | 44 |  |
| Female | 8 | 13 |  |
| Age |  |  | 1.000 |
| <50 | 5 | 13 |  |
| ≥50 old | 15 | 44 |  |
| Type |  |  | 0.912 |
| Squamous cell carcinoma | 12 | 30 |  |
| Small cell lung cancer | 0 | 5 |  |
| Adenocarcinoma | 7 | 19 |  |
| Others | 1 | 3 |  |
| Differentiation |  |  | 0.238 |
| High | 7 | 12 |  |
| Medium and low | 13 | 45 |  |

Chi square was used for significant difference test and p>0.05 indicates no statistical significance
